# A Two-Dimensional Multiple-Choice Model Accounting for Omissions

**DOI:** 10.3389/fpsyg.2018.02540

**Published:** 2018-12-11

**Authors:** Rodrigo Schames Kreitchmann, Francisco José Abad, Vicente Ponsoda

**Affiliations:** Department of Social Psychology and Methodology, Faculty of Psychology, Universidad Autónoma de Madrid, Madrid, Spain

**Keywords:** item response theory, multiple-choice items, polytomous responses, missing data, non-ignorable missing data, non-responses, omitted responses, guessing

## Abstract

This paper presents a new two-dimensional Multiple-Choice Model accounting for Omissions (MCMO). Based on Thissen and Steinberg multiple-choice models, the MCMO defines omitted responses as the result of the respondent not knowing the correct answer and deciding to omit rather than to guess given a latent propensity to omit. Firstly, using a Monte Carlo simulation, the accuracy of the parameters estimated from data with different sample sizes (500, 1,000, and 2,000 subjects), test lengths (20, 40, and 80 items) and percentages of omissions (5, 10, and 15%) were investigated. Later, the appropriateness of the MCMO to the Trends in International Mathematics and Science Study (TIMSS) Advanced 2015 mathematics and physics multiple-choice items was analyzed and compared with the Holman and Glas' Between-item Multi-dimensional IRT model (B-MIRT) and with the three-parameter logistic (3PL) model with omissions treated as incorrect responses. The results of the simulation study showed a good recovery of scale and position parameters. Pseudo-guessing parameters (*d*) were less accurate, but this inaccuracy did not seem to have an important effect on the estimation of abilities. The precision of the propensity to omit strongly depended on the ability values (the higher the ability, the worse the estimate of the propensity to omit). In the empirical study, the empirical reliability for ability estimates was high in both physics and mathematics. As in the simulation study, the estimates of the propensity to omit were less reliable and their precision varied with ability. Regarding the absolute item fit, the MCMO fitted the data better than the other models. Also, the MCMO offered significant increments in convergent validity between scores from multiple-choice and constructed-response items, with an increase of around 0.02 to 0.04 in *R*^2^ in comparison with the two other methods. Finally, the high correlation between the country means of the propensity to omit in mathematics and physics suggests that (1) the propensity to omit is somehow affected by the country of residence of the examinees, and (2) the propensity to omit is independent of the test contents.

## 1. Introduction

Missing responses occur frequently in educational and psychological assessments. Traditional psychometric models were originally designed to be used with complete data and therefore their application with missing data may provide biased parameter estimates (de Ayala et al., 2001; Finch, 2008). The size of these biases depends mostly on the proportion of missing data and the *ignorability* of the underlying missing data mechanisms.

Let **Y** = (*y*_*ij*_) denote a matrix of item response variables, where *y*_*ij*_ represents the response of the *i*th subject to item *j*. Let **Y**_obs_ correspond to the observed values of **Y**, and **Y**_mis_ the hypothetical values of the missing elements. Define a matrix **M** = (*m*_*ij*_) of missing data indicators, where *m*_*ij*_ = 1 if *y*_*ij*_ is missing and *m*_*ij*_ = 0 otherwise. Three underlying missing mechanisms can be derived from the dependencies between **Y** and **M** (Little and Rubin, 2002). If *P*(**M**|**Y**) = *P*(**M**) for all **Y**, the missing data are said to occur completely at random (MCAR). If *P*(**M**|**Y**) = *P*(**M**|**Y**_obs_) for all **Y**_mis_, the data are missing at random (MAR). Otherwise, if missing indicators depend on the unobserved part of the **Y**, the data are missing not at random (MNAR).

Under the IRT framework, the response functions for **Y** are assumed to be governed by a latent variable (θ) representing an examinees' ability, knowledge, attitude, etc. Similarly, **M** may depend on an examinee-specific parameter ξ characterizing a latent variable (e.g., propensity to skip, or speed in timed tests). Missing data are *ignorable* (i.e., correct inferences about θ can be drawn from **Y**_obs_) whenever mechanisms are MCAR or MAR and the traits involved in both processes are distinct - i.e., their joint parameter space is the product of the parameter spaces of each of them alone (Mislevy and Wu, 1996; Little and Rubin, 2002). On the other hand, if missing data satisfy MNAR or θ and ξ are not distinct, correct inferences about θ can only be made by modeling **M** and its relationship with **Y**.

Three major classes of *missingness* are routinely observed in psychological and educational assessments: (1) items not administered, (2) items not reached in a timed testing, and (3) omitted responses (e.g., Mullis et al., 2016; OECD, 2016). The first type usually results from applying different test forms and is *ignorable* in adaptive testing or when booklets are randomly assigned (Mislevy and Wu, 1996). Otherwise, if the forms are assigned based on educational or demographic variables, this information should be included in the model to allow for correct Bayesian estimates (Mislevy and Wu, 1996). Not reached non-responses are *ignorable* if the examinees did not interact meaningfully with the items not reached, had no information about their difficulties, and ξ, i.e., speed, and θ are distinct. If ξ and θ are not distinct, Bayesian inferences from **Y**_obs_ may be compromised unless the joint distribution of θ and ξ is considered in the estimation (Mislevy and Wu, 1996). Finally, omissions are consensually understood as the examinees' intentional non-responses (e.g., Budescu and Bar-Hillel, 1993; Mislevy and Wu, 1996; Bereby-Meyer et al., 2002; Rose, 2013; Budescu and Bo, 2015). It is reasonable to assume that the examinees are willing to optimize their outcomes (e.g., to be selected for a job, or to pass an exam) by maximizing their test scores. Therefore, the decision to omit may depend on their perceived gain or loss for responding to each item. In this case, skipping may depend on the probability of answering an item correctly, which configures MNAR, and the dependencies between **Y** and **M** should be addressed (Mislevy and Wu, 1996). This article focuses on the omitted responses and their possible effects on the parameter estimates.

Missing data treatments can be organized into three major types: (1) treated as *ignorable*, (2) data augmentation, and (3) modeling missing data. The first consists of ignoring non-responses, such as traditional IRT and factor analysis models, which accounts only for **Y**_obs_ and will provide inaccurate estimates if the *ignorability* criteria do not hold. The second class of treatments consists of assigning artificial realized values for **Y**_mis_, based either on deterministic preconceptions about the relationship between **M** and **Y** (e.g., recoding omissions as incorrect) or on model-based inferences from **Y** (e.g., item or person mean substitution, multiple imputation). Although extensively applied, deterministic imputation methods often make assumptions that are hardly acceptable considering the current missing data theory (Mislevy and Wu, 1996; Rose, 2013). Methods such as treating omissions as incorrect assume that the expected success probability for a non-response is the same as for responding incorrectly, which may be unrealistic. If the imputed dataset is modeled using the 3PL, for example, it has no theoretical sense to assume that the less proficient examinees omit with a probability defined by the pseudo-guessing parameter associated with the plausibility of the options.

Model-based augmentation methods, like multiple imputation, can be extremely useful for data with MCAR and MAR (Huisman and Molenaar, 2001; Finch, 2008, 2010). However, its use in MNAR situations may not be appropriate, given that non-responses are usually imputed with plausible values based on models that assume the *ignorability* of omissions. Unless the theory underlying the imputation procedure correctly represents the relationship between **Y** and **M**, the parameter estimates from the imputed dataset may be biased.

A variety of model-based treatments for non-ignorable missing data have been proposed in recent years (e.g., Holman and Glas, 2005; Okumura, 2014; Pohl et al., 2014; Debeer et al., 2017; Rose et al., 2017). As an example, the Symmetric Pattern Models (O'Muircheartaigh and Moustaki, 1999) predict the outcomes as a result of two steps: (1) to respond or skip, and (2) to select a specific answer when a response is provided. The model is approached by factorizing the likelihood function $P\left({\text{y}}_{i}^{\text{obs}},{\text{m}}_{i}|\theta_{i},\xi_{i}\right)=\prod_{j=1}^{J}P\left(y_{ij}^{\text{obs}},m_{ij}|\theta_{i},\xi_{i}\right)$ into $\prod_{j=1}^{J}P\left(y_{ij}^{\text{obs}}|m_{ij},\theta_{i}\right)P\left(m_{ij}|\xi_{i}\right)$, where ${\text{y}}_{i}^{\text{obs}}$ denotes the observed response pattern of the *i*th examinee. **M** is included as pseudo-items, so the model is analogous to a between-item multi-dimensional model, where **M** is governed by ξ and **Y**_obs_ by θ.

Several models derive from O'Muircheartaigh and Moustaki's definition. Holman and Glas (2005), for example, reformulate it to freely estimate the covariance between latent variables and present four equivalent forms with different parametrizations. The simplest form is the most used, and it is equivalent to a Between-item Multi-dimensional IRT model (B-MIRT). The B-MIRT model sets two measurement models, one for the observed responses (**Y**_obs_, where non-responses are coded as NA) and one for the omitted response indicators (**M**, where *m*_*ij*_ = 1 if *y*_*ij*_ = NA and 0 otherwise). Each measurement model is specified either as a Rasch or a 2PL model, where the ability governs the answering process underlying **Y**_obs_ and the propensity to skip governs the responding/not-responding process in **M** (Holman and Glas, 2005). The joint model is represented in Equation 1.

(1) $$\begin{array}{l} P\left(y_{i}^{obs},m_{i}|\theta_{i},\xi_{i},\beta ,\delta \right)=P\left(y_{i}^{obs}|m_{i},\theta_{i},\beta \right)P\left(m_{i}|\xi_{i},\delta \right) \end{array}$$

where **β** and **δ** denote the sets of structural parameters under the 2PL for the a given item and its associated pseudo-item in **M**, respectively. The dependency between the two measurement models is addressed through the correlation between θ and ξ. Similarly, De Boeck and colleagues approach this formulation through IRTree models (De Boeck and Partchev, 2012; Jeon and De Boeck, 2016; Debeer et al., 2017), which are mathematically equivalent to the B-MIRT (Debeer et al., 2017, p. 341).

Approaches based on the factorization of $P\left({\text{y}}_{i}^{\text{obs}},{\text{m}}_{i}|\theta_{i},\xi_{i}\right)$ are elegant in their simplicity and flexibility, given their broad assumptions about the relationship between **Y** and **M**. Nevertheless, the psychological processes underlying missing data mechanisms are not explicitly addressed and some definitions may not be accurate if considered from a psychological perspective. First, by using models from the logistic family for $P\left(y_{ij}^{\text{obs}}|{m_{ij},\theta }_{i}\right)$, the probability of success is asymptotic to zero as the proficiency decreases. In multiple-choice items this assumption can be unrealistic given that the examinees with low ability may attempt to guess their responses. By not accounting for guessing, a correct guess would be attributed to having a certain level of knowledge, which may lead to overestimated abilities of the less proficient examinees. Secondly, the events of responding/skipping (**m**_*j*_) are considered to be conditionally independent of choosing an answer once a response is provided (${\text{y}}_{j}^{\text{obs}}$), which is contradictory to the assumption that the examinees decide to omit depending on their probability of responding correctly.

Conversely, Lord's (1983) model for binary scored multiple-choice items with omissions makes explicit assumptions abouth the behavioral rationale for the omissions. The response process including omissions is defined by a combination of four subprocesses. Firstly, it is considered that an examinee can either prefer one of the alternatives with probability *R*(θ_*i*_) or be totally undecided. If one alternative is preferred, a response is made with $P^{*}\left(\theta_{i}\right)$ of being correct, which is monotonically increasing with ability. On the contrary, if the examinees have no preference, they will either omit with a probability *w*_*i*_ or guess at random with a probability to succeed which is reciprocal to the number of alternatives (*K*). Therefore, the overall probability of the *i*th examinee responding correctly is $P^{*}\left(\theta_{i}\right)\left[1-R\left(\theta_{i}\right)\right]+R\left(\theta_{i}\right)\left(1-w_{i}\right)K^{-1}$, while the probability of responding incorrectly is $[1-P^{*}\left(\theta_{i}\right)][1-R\left(\theta_{i}\right)]+R\left(\theta_{i}\right)\left(1-w_{i}\right)\left(1-K^{-1}\right)$, and omitting is *R*(θ_*i*_)*w*_*i*_.

Abad et al. (2009) presented a multi-group uni-dimensional model to account for omissions under a similar rational behavior perspective. Their model is based on the Multiple-Choice Model (MCM; Samejima, 1979; Thissen and Steinberg, 1984) and differs from Lord's (1983) formulation in some theoretical aspects. Firstly, having a preference and choosing an alternative are not considered as separate events. Instead, the probability function of the former is set to depend on the parameters defined for the probability function of the latter step (i.e., discrimination and difficulty). Secondly, it assumes that the distractors may be attractive for subjects with different proficiency levels, so their relationships with ability could be better described by a polytomous model, rather than a binary model. And thirdly, instead of estimating a probability *w* for each subject, the variability in the propensity to omit is addressed by dividing subjects into supposedly homogenous groups given the empirical proportions of omissions vs. errors.

This paper presents a new model to address some common features to multiple-choice items: (1) responses based on partial knowledge, (2) guessing behaviors, and (3) omitted responses. The new two-dimensional multiple-choice model accounting for omissions (MCMO) derives from the traditional Multiple-Choice Model (Samejima, 1979; Thissen and Steinberg, 1984) and the one proposed by Abad et al. (2009). However, the propensity to omit is included as a characteristic of the examinee, rather than a group variable. Firstly, a brief overview of the traditional Multiple-Choice Model is provided. After which, the model extensions made in this article are presented. Two studies were conducted to investigate the psychometric properties of the new model. A Monte Carlo simulation was carried out to analyze the accuracy of the MCMO estimates with different sample sizes, test lengths and the expected percentages of omitted responses. Finally, an illustration of the application of the new model with two subsets from TIMSS Advanced 2015 data (Mullis et al., 2016) is presented.

## 2. The Multiple-Choice Model

The MCM combines aspects of the three-parameter logistic (3PL) model (Lord, 1980) and the nominal response model (NRM; Bock, 1972). Like in the 3PL model, the MCM accounts for the guessing responses by allowing a non-zero left asymptote for the correct response. As in the NRM, it models polytomous responses and it assumes that the information provided by the distractors can be valuable for the estimation of ability because they may attract examinees with different ability levels. In the MCM, a *don't know* (DK) latent response state is included, representing the examinees who have no idea of the correct answer. On the contrary, thinking that one of the *K* item alternatives is correct is represented as being in a latent *know* state. For any given item, the probability of being in the *v*^th^ latent state is modeled by the NRM (Equation 2), where *v* ∈ {0, 1, ··· , *K*} and 0 denotes the DK state.

(2) $$\begin{array}{l} T\left(u_{i}=v|\theta_{i},\lambda \right)=\frac{exp\left(a_{v}\theta_{i}+c_{v}\right)}{\sum_{h=0}^{K}exp\left(a_{h}\theta_{i}+c_{h}\right)} \end{array}$$

where **λ** is a vector of *K* + 1 pairs of scale and position parameters associated with each latent state, *h* ∈ {0, 1, ··· , *K*}, and *u* denotes the latent states. The order of the scale parameters *a* is related to the degree of *correctness* of the latent states. The highest *a* value is expected to occur for the state related to the correct option and its latent response function should increase with ability. The order of the position parameters *c* reflects the relative predominance of each latent state at θ = 0.

The probability of the *i*th examinee selecting an alternative *k* in any given item is the sum of the probability of thinking that option *k* is correct, and the probability of guessing *k* (*k*:1, ··· , *K*) given he/she is in the *don't know* state (Equation 3). The term *d*_*k*_ denotes an item-specific pseudo-guessing parameter representing the plausibility of an alterative *k* for the examinees in DK. Let **γ** = (**λ**, *d*_1_, ··· , *d*_*K*_):

(3) $$\begin{array}{l} P\left(x_{i}=k|\theta_{i},\gamma \right)=T\left(u_{i}=k|\theta_{i},\lambda \right)+T\left(u_{i}=0|\theta_{i},\lambda \right)d_{k} \end{array}$$

The MCM, as well as the MCMO, that it will presented next, can also be implemented with dichotomously scored responses (with distractors recoded into a single category), which should offer comparable results if the latent states associated with the incorrect alternatives have similar discrimination parameters. In this case, the *d* parameter for the collapsed incorrect category would represent the overall probability of failing by guessing in the DK state.

## 3. A Multiple-Choice Model for Omissions

The Multiple-Choice Model for Omissions extends the MCM by assuming that omissions also reflect being in the *don't know* state (Equation 4). However, the decision about whether to guess, or to omit, in this latent state depends on the latent propensity to omit ξ distinct from θ (Equation 5). It should be noted from Equation 4 that the MCMO specifies a non-ignorable missing data mechanism even if θ and ξ are uncorrelated, given that the probability of omitting depends on being in the DK state, which is governed by the ability.

(4) $$\begin{array}{l} P\left(x_{i}=0|\theta_{i},\xi_{i},\lambda \right)=T\left(u_{i}=0|\theta_{i},\lambda \right)w_{i} \end{array}$$

where *x*_*i*_ = 0 represents the omission outcome for the *i*th subject.

(5) $$\begin{array}{l} w_{i}=P\left(x_{i}=0|u_{i}=0,\xi_{i}\right)=\frac{1}{1+exp\left(-\xi_{i}\right)} \end{array}$$

Complementarily, selecting an alternative *k* may occur either if an examinee thinks *k* is correct or if he/she does not know the answer, decides to guess rather than to omit, and guesses *k* with probability *d*_*k*_ (Equation 6).

(6) $$\begin{array}{l} P\left(x_{i}=k|\theta_{i},\xi_{i},\gamma \right)=T\left(u_{i}=k|\theta_{i},\lambda \right)+T\left(u_{i}=0|\theta_{i},\lambda \right)\left(1-w_{i}\right)d_{k} \end{array}$$

**Figure 1:**
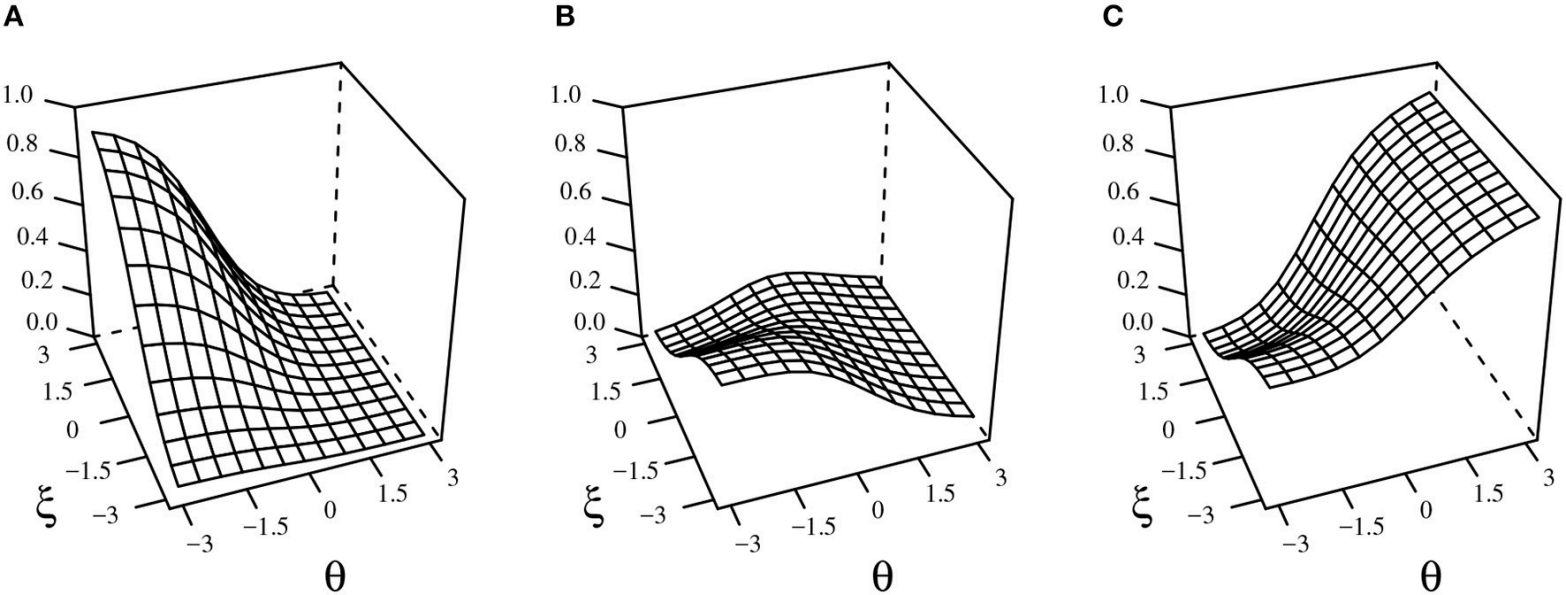
Example of the MCMO functions for omitted **(A)**, incorrect **(B)**, and correct **(C)** responses. γ = (*a*_0_ = −1.5, *a*_1_ = −0.5, *a*_2_ = 0, *c*_0_ = −0.2, *c*_1_ = −0.9, *c*_2_ = 0, *d*_1_ = 0.5, *d*_2_ = 0.5).

As shown in Figure 1, the MCMO is not a traditional compensatory or non-compensatory multi-dimensional model. In the *omission* ORF (Figure 1A), as the ability decreases and the propensity to omit increases, the probability of omitting approaches its maximum. In turn, the probability of selecting an incorrect option grows when both the ability and the propensity to omit decrease. Figure 1A also shows that when the propensity to omit is low, examinees with average ability levels tend to select an incorrect option based on partial or wrong information. Finally, the *correct* ORF (Figure 1C) increases globally together with ability. However, for low θ values, the probability of responding correctly will be asymptotic to *d*_*k*_ when ξ is low and to zero when ξ is high. Also, as can be inferred from Equations 4 to 6, the ORFs relative to the alternatives will asymptotically approach either the NRM or the MCM as ξ gets higher or lower, respectively.

It should be noted that the term *T*(*u*_*i*_ = *k*|θ_*i*_, **λ**) in Equation 6 can also be written as *T*(*u*_*i*_ = *k*|*u*_*i*_ ≠ 0, θ_*i*_, **λ**)*T*(*u*_*i*_ ≠ 0|θ_*i*_, **λ**). From this formulation, it is possible to see that the MCMO and the Lord's model (1983) share similar general definitions when the responses are dichotomously scored, where *T*(*u*_*i*_ = *K*|*u*_*i*_ ≠ 0, θ_*i*_, **λ**), with *K* being the correct alternative, is analogous to $P^{*}\left(\theta_{i}\right)$, and *T*(*u*_*i*_ = 0|θ_*i*_, **λ**) to *R*(θ_*i*_). However, the two models differ in three important aspects: (1) the guessing probability when in the DK/no-preference state is freely estimated in the MCMO while fixed to *K*^−1^ in Lord's model; (2) under the MCMO, there is no possibility of guessing when in a *know* state, while the Lord's model allows guessing also when a preference is felt, by specifying $P^{*}\left(\theta_{i}\right)$ under the 3PL model; and finally (3) in Lord's model, $P^{*}\left(\theta_{i}\right)$ and *R*(θ_*i*_) are not explicitly linked, while in the MCMO the probability *T*(*u*_*i*_ = 0|θ_*i*_, **λ**) depends on the characteristics of the alternatives (see Equation 2), which is more consistent with the psychological theory of omissions.

### 3.1. Identification

For the model to be identified, either the *a* and *c* parameter of one of the latent states must be fixed to an arbitrary value, or the constraint ∑*a*_*h*_ = ∑*c*_*h*_ = 0 should be imposed. Moreover, opposite *a* values could yield equal ORFs by flipping the ability trait. In order to set θ “right-side-up,” attributing lower initial values to the *a* parameters related to the DK state and the incorrect categories should be sufficient (Thissen and Steinberg, 1984). Also, the ability distribution is set to *N*(0, 1).

### 3.2. Parameter Estimation

Item parameters can be estimated using either marginal maximum likelihood or Bayesian marginal maximum likelihood by applying Bock and Aitkin's two-step EM-like procedure (Bock and Aitkin, 1981). Similarly to the algorithm proposed by Dempster et al. (1977), it consists of an iterative procedure with Expectation and Maximization steps until the changes of the estimates between the iterations are negligible. The parameters of the distribution of ξ are estimated from the posterior expectations generated at the E-step and updated with every iteration, and the covariance between the traits is set to 0. The script for the estimation of the MCMO using the *mirt* package (Chalmers, 2012) in R (R Core Team, 2017) is provided in the Supplementary Material.

The pseudo-guessing parameters *d*_*k*_ can be bound to the probability metric and constrained to $\overset{K}{\underset{h=1}{\sum }}d_{h}=1$ by using the transformation in Equation 7, where *d*′ is estimated instead (Thissen and Steinberg, 1984). Given that only a few subjects are expected at the low extreme of the ability distribution, the data can be little informative about *d*′ and using informative priors may benefit the estimation.

(7) $$\begin{array}{l} d_{k}=\frac{exp\left(d_{k}^{\prime }\right)}{\sum_{h=1}^{K}exp\left(d_{h}^{\prime }\right)} \end{array}$$

Person parameters can be estimated with the *Expected A Posteriori* (EAP) method by assuming the estimated structural parameters as true.

## 4. Simulation Study

A simulation study was conducted to evaluate the parameter recovery with different sample sizes, test lengths and proportions of omitted responses.

### 4.1. Methods

#### 4.1.1. Data Generation

Twenty-seven conditions were simulated by combining three sample sizes, *N* = (500, 1000, 2000), three test lengths, *J* = (20, 40, 80), and three expected percentages of omitted responses, $\bar{p}O=\left(5\%,10\%,15\%\right)$. The item responses were generated with three alternatives and one omission category using the MCMO functions specified in Equations 4 to 6. One hundred replications were carried out for each condition. The true values of the ability and the propensity to omit were drawn from two independent standard normal distributions.

The item parameters related to the correct alternative were fixed to zero to enable identifiability. The values of the two free *d*′ parameters were drawn from *N*(0, 0.5), so that $E\left[d_{k}\right]=K^{-1}$. The three free scale parameters (i.e., *a*_0_, *a*_1_, and *a*_2_) were drawn from a multivariate normal distribution with μ_*a*_ = (−2, −1, −1), σ_*a*_ = 1 and covariances of 0.65, so the expected values of the parameters were ordered by *correctness*. The two free position parameters of the *know* states related to the incorrect alternatives (i.e., *c*_1_ and *c*_2_) were drawn from *N*(−0.5, 1), while *c*_0_*N*(μ_*c*_0__, 1). The expected percentage of omitted responses for each dataset was generated by manipulating the predominance of the *don't know* state probability by shifting the mean of the DK position parameter true distribution (μ_*c*_0__). As presented in Equation 4, by increasing the expected probability of being in the DK state, the expected probability of omitting increases linearly. Given the known true distributions of the model parameters, the values of μ_*c*_0__ were chosen to satisfy the condition $E\left[P\left(x=0|\theta ,\xi ,\gamma \right)\right]=100^{-1}\bar{p}O$, where *E*[*P*(*x* = 0|θ, ξ, γ)] was given by:

(8) $$\begin{array}{l} E\left[P\left(x=0|\theta ,\xi ,\gamma \right)\right]=\int \cdot \cdot \cdot \int P\left(x=0|\theta ,\xi ,\gamma \right)g\left(\theta \right)g\left(\xi \right)g\left(\phi \right)\\ d\theta d\xi d\phi_{1}\cdot \cdot \cdot d\phi_{s} \end{array}$$

where **ϕ** denotes the set of estimated parameters, i.e., $a_{0},\ldots ,a_{3},c_{0},\ldots ,c_{3},d_{1}^{\prime },d_{2}^{\prime }$, and sub-index *s* represents the number of estimated item parameters. The values of μ_*c*_0__ that satisfied the conditions with 5,10, and 15% of omissions were -2.10, -0.99, and -0.22, respectively.

#### 4.1.2. Parameter Estimation

The item parameters were estimated using Bayesian marginal maximum likelihood with the EM algorithm (Bock and Aitkin, 1981) implemented by the *mirt* package (Chalmers, 2012) in *R* (R Core Team, 2017). Prior distributions of *N*(0, 0.5) were set for the parameters *d*′ of the incorrect alternatives. Person parameters were estimated with EAP also using the *mirt* package. The MCMO estimation routine is provided in the Supplementary Material.

#### 4.1.3. Recovery of Model Parameters

The accuracy of the parameter estimates was assessed through three indicators: (1) the correlation between the true and estimated parameters ($\rho_{\delta \hat{\delta }}$, where δ denotes the true parameter being analyzed and $\hat{\delta }$ the estimate of δ), (2) the mean error (ME; Equation 9), and (3) the root-mean-square error (RMSE; Equation 10), where *N* represents the number of observations in each replica (*N* reflects the test length if δ is an item parameter and the sample size if δ is a person parameter), and *R* denotes the number of replications (i.e., *R* = 100). The correlation coefficient indicates the linearity between the estimated and the true parameter values, while the ME summarizes the average estimate bias. The RMSE is a broader measure of accuracy affected by both $\rho_{\delta \hat{\delta }}$ and ME (Roberts and Laughlin, 1996, p. 243). For ease of presentation, the $\rho_{\delta \hat{\delta }}$, ME and RMSE of the parameters associated with the *know* latent states were averaged within each parameter type, i.e., scale, position or pseudo-guessing.

(9) $$\begin{array}{l} \text{ME}\left(\delta \right)=\frac{1}{R}\overset{R}{\underset{r=1}{\sum }}\frac{\sum_{n=1}^{N}\left({\hat{\delta }}_{nr}-\delta_{nr}\right)}{N} \end{array}$$

(10) $$\begin{array}{l} \text{RMSE}\left(\delta \right)=\frac{1}{R}\overset{R}{\underset{r=1}{\sum }}\sqrt{\frac{\sum_{n=1}^{N}{\left({\hat{\delta }}_{nr}-\delta_{nr}\right)}^{2}}{N}} \end{array}$$

The difficulty of estimating the pseudo-guessing parameters with realistic sample sizes is well-known to occur given the small number of subjects with extreme ability levels. Given that the EAP method assumes item parameters estimates as true, and that response probabilities close to *d* are expected for the less proficient subjects, the inaccuracy of *d* may affect the estimation of low abilities. A regression model was fitted to investigate such effect. For each simulated dataset, the average RMSE of *d*′ was set as a predictor of the RMSE of the simulees with the 20% lowest θ values. The proportion of variance explained (*R*^2^) is presented.

Furthermore, as the omissions are conditional on being in the DK, the information about ξ should also depend on θ, and the overall RMSE, the ME and the correlation coefficient may not represent the estimation error across all the latent space. To depict the variation in the estimation errors of ξ across θ, the three accuracy indicators were also presented separately for the subjects with the 20% lowest and the 20% highest true ability values.

#### 4.1.4. Recovery of Expected Response Functions

Given the complexity of the model, the uncertainty of the individual item parameter estimates does not necessarily imply any inaccuracy in the expected functions. The root-integrated-square error (RISE) indicates the expected discrepancy between the estimated and the true expected option response functions (ORF) given a known latent trait distribution. The RISE for the *h*th category of each item (Equation 11) was approximated through eleven quadrature points from -3 to 3 for each latent trait, resulting in 121 quadrature combinations.

(11) $$\begin{array}{l} {\text{RISE}}_{h}=\sqrt{\iint {\left[P\left(x=h|\theta ,\xi ,\hat{\gamma }\right)-P\left(x=h|\theta ,\xi ,\gamma \right)\right]}^{2}g\left(\theta \right)g\left(\xi \right)d\theta d\xi } \end{array}$$

The RISE results were then averaged within the types of response functions, i.e., omission, incorrect, and correct responses, and replications.

Finally, ANOVAs were conducted to summarize the effect of the sample size, test length and expected percentage of omitted responses over each accuracy indicator. Given the high number of datasets generated, most of the main effects are expected to be significant (*p* < 0.05). Therefore, only the high partial eta-squared effect sizes (η^2^ ≥ 0.14) will be discussed.

### 4.2. Results

#### 4.2.1. Data Generation

The average percentage of omissions obtained in the simulated datasets were 5.1, 9.8, and 14.7% for the three $\bar{p}O$ conditions, respectively.

#### 4.2.2. Recovery of Item Parameters

The ANOVA main effect sizes are shown in Table 1. The interaction effects ranged from very small to slightly moderate and are not shown. Regarding the RMSE and the correlation coefficient, the recovery of scale and position parameters was affected primarily by the sample size and the percentage of omissions.

**Table 1:** ANOVA main effect sizes for the accuracy measures of the parameter estimates.

	**Root-mean-square error**			**Pearson correlation**			**Mean error**		
**Parameter**	**$\eta_{N}^{2}$**	**$\eta_{J}^{2}$**	**$\eta_{\bar{p}O}^{2}$**	**$\eta_{N}^{2}$**	**$\eta_{J}^{2}$**	**$\eta_{\bar{p}O}^{2}$**	**$\eta_{N}^{2}$**	**$\eta_{J}^{2}$**	**$\eta_{\bar{p}O}^{2}$**
*a*_0_	**0.42**	0.05	0.07	**0.35**	0.03	0.09	**0.15**	0.02	0.01
ā_*k*_	**0.33**	0.01	**0.23**	**0.25**	0.01	**0.19**	0.03	0.00	0.00^*^
*c*_0_	**0.24**	0.01	**0.14**	**0.33**	0.00	**0.27**	0.02	0.00^*^	0.05
${\bar{c}}_{k}$	**0.17**	0.00^*^	**0.18**	**0.22**	0.00^*^	**0.26**	0.07	0.00	0.07
*d*′	**0.20**	0.04	**0.29**	**0.35**	0.13	**0.46**	0.00^*^	0.00^*^	0.00^*^
θ	0.10	**0.94**	**0.40**	0.04	**0.92**	**0.34**	0.01	0.00	0.00
ξ	0.08	**0.77**	**0.75**	0.01	**0.88**	**0.88**	0.00^*^	0.00^*^	0.00^*^
ξ_*lowθ*_	0.04	**0.72**	**0.59**	0.01	**0.79**	**0.70**	0.00	0.03	0.01
ξ_*highθ*_	0.02	**0.35**	**0.37**	0.00	**0.54**	**0.61**	0.00	0.01	0.00

*N, sample size; J, test length; $\bar{p}O$, percentage of omissions; ξ_lowθ_, Recovery of ξ for the subjects with 20% lowest ability levels; ξ_highθ_, Recovery of ξ for the subjects with 20% highest ability levels*;

* *Non-significant effects. Boldfaced, high effect sizes (η^2^ ≥ 0.14)*.

As shown in Table 2, the linearity between the true and estimated scale and position parameters was high for all the conditions. On the other hand, the pseudo-guessing parameters had the lowest correlation coefficients, with a minimum of 0.33 and a maximum of 0.56, and RMSE ranging from 0.27 to 0.31. The effect of $\bar{p}O$ acted in different directions for the parameters associated with the *don't know* state and with the *know* states. As the $\bar{p}O$ increased, the parameters of the DK state were better estimated, which occurs because the omitted responses are especially informative about DK. On the other hand, as the proportion of omissions increased, the available information about the *know* states diminished and their scale and position parameter estimates were less precise.

**Table 2:** Accuracy of the parameter estimates given the sample sizes, test lengths and the percentages of omissions.

	**Sample size**			**Test length**			**Percentage of omissions**		
**Parameter**	**500**	**1,000**	**2,000**	**20**	**40**	**80**	**5%**	**10%**	**15%**
**ROOT-MEAN-SQUARE ERROR**									
*a*_0_	0.59	0.37	0.25	0.45	0.39	0.36	0.46	0.36	0.38
ā_*k*_	0.46	0.30	0.20	0.35	0.32	0.30	0.23	0.30	0.43
*c*_0_	0.60	0.35	0.23	0.43	0.37	0.37	0.55	0.33	0.30
${\bar{c}}_{k}$	0.58	0.34	0.22	0.39	0.37	0.37	0.22	0.33	0.58
*d*′	0.31	0.29	0.27	0.30	0.29	0.28	0.31	0.29	0.27
θ	0.33	0.32	0.32	0.42	0.32	0.24	0.31	0.32	0.34
ξ	0.82	0.80	0.79	0.90	0.81	0.71	0.90	0.80	0.70
ξ_*lowθ*_	0.65	0.63	0.62	0.76	0.63	0.50	0.74	0.62	0.54
ξ_*highθ*_	0.95	0.94	0.93	0.99	0.95	0.88	1.00	0.94	0.88
**PEARSON CORRELATION**									
*a*_0_	0.88	0.94	0.97	0.92	0.93	0.94	0.91	0.94	0.94
ā_*k*_	0.91	0.95	0.98	0.94	0.95	0.95	0.97	0.95	0.92
*c*_0_	0.90	0.96	0.98	0.94	0.95	0.95	0.91	0.96	0.97
${\bar{c}}_{k}$	0.91	0.95	0.98	0.95	0.95	0.94	0.98	0.96	0.90
*d*′	0.35	0.46	0.54	0.40	0.46	0.50	0.33	0.46	0.56
θ	0.94	0.94	0.94	0.91	0.95	0.97	0.95	0.94	0.94
ξ	0.59	0.60	0.60	0.48	0.60	0.71	0.48	0.61	0.70
ξ_*lowθ*_	0.77	0.78	0.78	0.68	0.78	0.87	0.70	0.79	0.84
ξ_*highθ*_	0.35	0.35	0.36	0.25	0.35	0.46	0.23	0.36	0.47
**MEAN ERROR**									
*a*_0_	-0.15	-0.08	-0.04	-0.11	-0.09	-0.07	-0.08	-0.08	-0.11
ā_*k*_	-0.03	-0.01	-0.01	-0.02	-0.02	-0.01	-0.02	-0.02	-0.02
*c*_0_	-0.05	-0.01	0.01	-0.01	-0.01	-0.03	-0.06	-0.01	0.02
${\bar{c}}_{k}$	-0.08	-0.03	-0.01	-0.05	-0.04	-0.04	-0.01	-0.03	-0.08
*d*′	0.00	0.00	0.00	0.00	0.00	0.00	0.00	0.01	0.00
θ	-0.01	0.00	0.00	0.00	0.00	0.00	0.00	0.00	0.00
ξ	-0.03	-0.03	-0.03	-0.03	-0.04	-0.02	-0.04	-0.03	-0.02
ξ_*lowθ*_	0.09	0.08	0.08	0.12	0.08	0.05	0.06	0.09	0.10
ξ_*highθ*_	-0.05	-0.05	-0.05	-0.06	-0.06	-0.03	-0.05	-0.05	-0.05
**ROOT-INTEGRATED-SQUARE ERROR**									
Omission	0.022	0.015	0.011	0.017	0.015	0.015	0.013	0.016	0.018
Incorrect	0.028	0.020	0.014	0.022	0.021	0.020	0.020	0.021	0.021
Correct	0.031	0.022	0.016	0.023	0.022	0.023	0.022	0.023	0.024

*ξ_lowθ_: Recovery of ξ for the subjects with 20% lowest ability levels; ξ_highθ_: Recovery of ξ for the subjects with 20% highest ability levels*.

With regard to the mean error indicators, the estimates of scale parameters associated with the DK state became slightly negatively biased as sample size decreased (η^2^ = 0.15). Interestingly, the recovery of *d*′ parameters improved with the increase of omitted responses indicating that these parameters are sensitive to the accuracy of the DK response function. An *R*^2^ < 0.01 was obtained for the regression analysis of the average accuracy of *d*′ over the accuracy of the lowest 20% of θ in each dataset. This result indicates that the precision of *d*′ had little effect on the recovery of the low θ values.

#### 4.2.3. Recovery of the Expected Response Functions

In general, the small root-integrated-square errors depicted in Table 2 indicate a good recovery of the three types of response functions, i.e., omission, incorrect and correct. The omission response function showed the highest accuracy and improved mainly with sample size (Table 3).

**Table 3:** ANOVA main effect sizes in the expected root-integrated-square error for each type of response function.

	**Root-integrated-square error**		
**ORF type**	**$\eta_{N}^{2}$**	**$\eta_{J}^{2}$**	**$\eta_{\bar{p}O}^{2}$**
Omission	**0.61**	0.11	**0.29**
Incorrect	**0.92**	**0.15**	0.09
Correct	**0.81**	0.03	0.06

*N, sample size; J, test length; $\bar{p}O$, percentage of omissions; Boldfaced, high effect sizes (η^2^ ≥ 0.14)*.

#### 4.2.4. Recovery of Person Parameters

The recovery of person parameters was highly affected by both test length and the percentage of omissions. However, the ability levels were accurate in all the conditions, with a minimum $\rho_{\theta \hat{\theta }}$ of around 0.91 up to 0.95, as test length increased (see Table 2). As the $\bar{p}O$ increased the RMSE of θ and the $\rho_{\theta \hat{\theta }}$ decreased slightly, although always staying above acceptable levels. The average accuracy of the propensity to omit was generally lower and depended on the test length, the proportion of omissions, and the level of ability of the simulees. As shown in Table 2, the accuracy of ξ was higher for the longer tests, for the greater $\bar{p}O$, and for the simulees with the lowest θ.

## 5. Empirical Study

This study investigates the appropriateness of the MCMO to multiple-choice response data from the Trends in International Mathematical and Science Study (TIMSS) Advanced 2015 assessment. TIMSS is a large-scale international study that provides comparative information about educational achievement across countries. Within this program, TIMSS Advanced aims to assess the advanced mathematics and physics achievements of students in their final year of secondary school.

Firstly, the reliability of the trait estimates was investigated. Secondly, the absolute fit of the MCMO was analyzed and compared with the fit provided by two alternative models, the Holman and Glas' Between-item Multi-dimensional IRT model (B-MIRT; Holman and Glas, 2005), and the 3PL model with an incorrect-answer substitution (3PL-IAS), which is used in the scoring procedure of TIMSS Advanced 2015. Thirdly, the evidence for the convergent validity of the ability estimates across multiple-choice and constructed-response formats was obtained. Finally, some observations on the propensity to omit under the MCMO were made.

### 5.1. Methods

#### 5.1.1. Data Description

The achievement data from TIMSS Advanced 2015 was divided into two datasets: one for mathematics, and one for physics. These datasets included populations from nine countries: France, Italy, Lebanon, Norway, Portugal, the Russian Federation, Slovenia, Sweden, and the United States (LaRoche and Foy, 2016). The testing design consisted of 6 booklets for mathematics and 6 for physics, with multiple-choice (MC), constructed-response (CR), and a few compound multiple-choice formats. MC items were made with either 4 or 5 alternatives, and the compound multiple-choice items consisted of sets of two-alternative interdependent items that were scored together given their count of correct responses. The samples that responded to the physics and mathematics items were independent (Martin et al., 2016).

In TIMSS Advanced 2015, there is no penalty for wrong answers and the examinees are encouraged to respond to all of the items. In its scoring procedure (Martin et al., 2016), omissions are treated as incorrect for both item and person parameter estimation. Multiple-choice item responses are recoded into correct/incorrect and estimated using the 3PL-IAS. Constructed-response items are assumed to follow the Generalized Partial Credit Model (Muraki, 1992) with omissions imputed as incorrect (GPCM-IAS).

One booklet was analyzed for each test content to avoid introducing the effects of the missing data derived from the testing design. Booklet 6 from the mathematics assessment, and Booklet 7 from the physics assessment were chosen as they contained the most items. Table 4 describes the datasets relative to each of the booklets used. The compound multiple-choice items were excluded from the analysis and not-reached responses were ignored.

**Table 4:** Descriptives for the TIMSS Advanced 2015 datasets used in the study.

	**Mathematics**	**Physics**
Booklet ID	6	7
MC items	21	18
CR items	12	12
% MC omitted	5.8	2.8
% MC not reached	1.9	0.2
% CR omitted	18.2	12.8
% CR not reached	0.1	0.8
*N*	5,966	4,078

#### 5.1.2. Model Estimation

For this study, the multiple-choice items were modeled under the MCMO using the polytomous responses, the B-MIRT and the 3PL-IAS. As in the simulation study, the structural parameters of the MCMO were estimated using Bayesian marginal maximum likelihood with the EM algorithm (Bock and Aitkin, 1981) implemented by the *mirt* package (Chalmers, 2012). The item parameters associated with the correct *know* state were fixed to zero and priors of *N*(0, .5) were set for the free *d*′ parameters.

The B-MIRT was specified as in 1 and its parameters were estimated using Marginal Maximum Likelihood with the EM algorithm (Bock and Aitkin, 1981) implemented by the *mirt* package (Chalmers, 2012).

The 3PL-IAS consists on the estimation of the 3PL model after re-scoring omissions as incorrect. The function of correct responses to an item under the 3PL model is traditionally formulated as in Equation (12):

(12) $$\begin{array}{l} P\left(x_{i}=1|\theta_{i},\beta ,c\right)=c+\frac{1-c}{1+\exp\left[-a\left(\theta_{i}-b\right)\right]} \end{array}$$

where **β** denotes the set of discrimination (*a*) and difficulty (*b*) parameters, and *c* represents the pseudo-guessing parameter associated with the correct category.

The parameters of the 3PL-IAS were estimated by the same method as the MCMO, using priors of *N*[logit(*K*^−1^), 0.5] for the logit of the pseudo-guessing parameters *c*. All person parameters were estimated using EAP.

The responses to the constructed-response items were modeled with both the GPCM-IAS, as in TIMSS scoring procedure, and the B-MIRT. The probabilities $P\left(y_{i}^{obs}|m_{i},\theta_{i},\beta \right)$ in the B-MIRT for constructed-response items with partially correct scores was specified with the Generalized Partial Credit Model (Muraki, 1992). Their parameters were also estimated using Marginal Maximum Likelihood with the EM algorithm (Bock and Aitkin, 1981) implemented by the *mirt* package (Chalmers, 2012). To distinguish the B-MIRT results for the different item formats in the empirical study, B-MIRT_*MC*_ will reffer to the one used with multiple-choice items, and B-MIRT_CR_ with constructed-response items.

#### 5.1.3. Trait Reliabilities Under the MCMO

Two types of reliability are provided. Firstly, the reliabilities conditional on the θ and ξ values (Equation 13) are represented graphically.

(13) $$\begin{array}{l} \rho_{\theta \theta^{\prime }}=1-\left(\frac{{SE}_{\theta }^{2}}{{\hat{\sigma }}_{\theta }^{2}}\right) \end{array}$$

The calculations were made using the asymptotic error variances, *SE*^2^, obtained from the diagonal of the inverse of the Bayesian Fisher Information matrix at several points in the latent space. The empirical reliabilities are also presented, calculated using the variances of the estimated person parameters, which were divided by the sum of their variances and the average of the squared standard error estimates (Equation 14).

(14) $$\begin{array}{l} {\bar{\rho }}_{\theta \theta^{\prime }}=\frac{\text{var}\left(\hat{\theta }\right)}{\text{var}\left(\hat{\theta }\right)+{\bar{SE}}_{\theta }^{2}} \end{array}$$

#### 5.1.4. Goodness-of-Fit

To avoid overfitting, the samples of mathematics and physics were randomly split into two sub-samples, one for parameter calibration and the other for cross-validation. The χ^2*^ fit index proposed by Stone and colleagues (Stone, 2000; Stone and Hansen, 2000; Stone and Zhang, 2003) was analyzed for each item. Considering that the true trait levels are unknown, the pseudocounts of each response category and trait value were obtained by numerically approaching the trait distribution through 11 gridpoints for each dimension (121 points in total) from -3 to 3 standard deviations from the means. The discrepancies between observed and expected pseudocounts at each grid point and response category were calculated using the traditional χ^2^ formula and summed to provide item-level fit statistics.

The χ^2*^ index follows a scaled chi-square distribution. As in Stone (2000), the scaling factors and χ^2*^ distribution parameters were approximated through a parametric bootstrap with 500 replications. The magnitude of the discrepancies was classified according to the χ^2*^/*df* ratio as either very small (<1), small (≥1 and <2), moderately large and (≥2 and <3), and large (≥3) (e.g., Drasgow et al., 1995; Chernyshenko et al., 2001).

#### 5.1.5. Validity of Ability Estimates

The convergent validities between the ability scores obtained from the multiple-choice and constructed-response items were analyzed. The increment on the convergent validity provided by the MCMO was approached through hierarchical linear regressions. For each test content, four linear regression models were fitted, using: (1) the 3PL-IAS scores, (2) both 3PL-IAS and MCMO scores, (3) the B-MIRT_MC_ scores, and (4) both B-MIRT_MC_ and MCMO scores, as independent variables. The dependent variables were the ability scores in the contructed-response items under either the GPCM-IAS or the B-MIRT_CR_. The *R*^2^, part correlations and *F*-test statistics of the change between models 1 and 2, and 3 and 4 are provided.

To investigate the common variance between the multiple-choice and constructed-response scores in different points of the ability trait, an approximation to the *R*^2^ at the examinee-level was calculated (Equation 15). It represents the contribution of each examinee to the total *R*^2^, where $\underset{i}{\sum }R_{i}^{2}=R^{2}$. A graphical representation of the average $R_{i}^{2}$ in seven ranges of the constructed-response ability estimates is presented.

(15) $$\begin{array}{l} R_{i}^{2}=\frac{{\left(f_{i}-ȳ\right)}^{2}}{\sum_{i}{\left(y_{i}-ȳ\right)}^{2}} \end{array}$$

where *f*_*i*_ is the fitted value for the *i*th examinee, *y*_*i*_ represents its observed value in the independent variable, i.e., GPCM-IAS scores, and ȳ is the mean of the independent variable.

Finally, given that the main contribution of the MCMO depends on the occurrence of omitted responses, the ability estimates for each of the three models were compared in five groups of examinees with proportion of omissions: 0, (0 - 0.1], (0.1 - 0.2], (0.2 - 0.3], (0.3 - maximum]. Also, to investigate if the ability estimates under the MCMO were representing the construct better than the other models in these groups, the incremental validity was also analyzed for these groups through the part correlations between the MCMO proficiency scores and the part of the constructed-response scores that were not explained by the 3PL-IAS or by the B-MIRT_MC_.

#### 5.1.6. Consistency of Propensity to Omit Estimates

The preliminary evidence for the consistency of the propensity to omit estimates in the MCMO was investigated. Given that an examinee only responded to one multiple-choice test in TIMSS Advanced 2015, either physics or mathematics, it was not possible to analyze directly the consistency of the propensity to when the test contents are different. Rather, this association was examined at the country-level, computing the correlation between the averages $\hat{\xi }$ in mathematics and physics across countries.

### 5.2. Results

The estimated distributions of the propensities to omit in multiple-choice items were approximately *N*(−2.65, 1.74) in mathematics and *N*(−3.22, 1.40) in physics, indicating that the expected probabilities of omitting in the DK state were 0.07 and 0.04, respectively. On average, the order of the scale parameters followed the theoretical expectations, with the most negative values for the *don't know* state and the highest for the *know* state associated with the correct alternative. The position parameters of DK were on average slightly higher than the others in both exams, indicating a small predominance of its probability when θ = 0.

#### 5.2.1. Trait Reliabilities Under the MCMO

**Figure 2:**
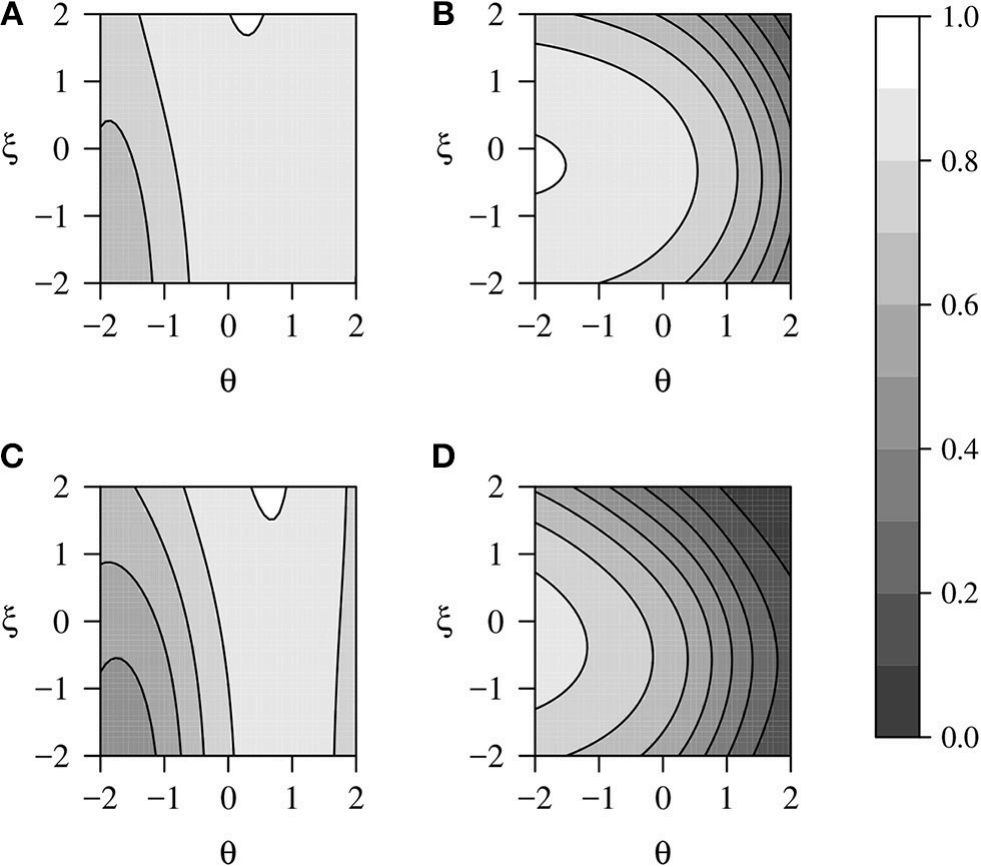
Expected point-reliabilities in TIMSS Advanced 2015 for **(A)** the ability in mathematics, **(B)** the propensity to omit in mathematics items, **(C)** the ability in physics, **(D)** the propensity to omit in physics items.

The empirical reliabilities were 0.80 for the mathematics scores, and 0.71 for the physics scores, and 0.60 and 0.41 for the propensity to omit in the mathematics and physics items, respectively. The lower reliability of ξ in the physics items may be due to the smaller number of omitted responses in this dataset, resulting in less available information about ξ. Although the empirical reliabilities of ξ were low in both tests, Figure 2 shows that there was a great variation in the conditional reliability across the latent space, with higher reliabilities for subjects with low abilities and central propensities to omit. This is to be expected since ξ only influences the responses when the subject is in a DK state, and this state is more probable at low θ values. For both exams, the conditional reliabilities of θ were higher for those examinees with medium θ levels and improved as ξ increased.

#### 5.2.2. Goodness-of-Fit

Table 5 shows the χ^2*^/*df* ratios for the MCMO, the B-MIRT_MC_ and the 3PL-IAS models. The χ^2*^/*df* ratios for the MCMO were below three in all items, indicating a good overall item-fit. By contrast, the 3PL-IAS provided the worst results, with five items highly misfitted in mathematics and six in physics. The fitness of the B-MIRT_MC_ was slightly worse than the MCMO. With regard to the mean of χ^2*^/*df*, the MCMO also seemed to have a qualitative advantage over the alternative models in both mathematics and physics.

**Table 5:** Frequencies, Means, and Standard Deviations (SDs) of the χ^2*^/*df* ratios for the multiple-choice items in TIMSS Advanced 2015.

	Frequency of χ^2*^/*df*					
**Test content and model**	**< 1**	**1− < 2**	**2− < 3**	**3**	**Mean**	**SD**
**Mathematics**						
MCMO	2	17	2	0	1.49	0.40
3PL-IAS	6	5	5	5	2.10	1.27
B-MIRT_MC_	0	11	8	2	2.12	0.83
**Physics**						
MCMO	1	14	3	0	1.63	0.45
3PL-IAS	3	6	3	6	2.32	1.32
B-MIRT_MC_	3	10	4	1	1.79	1.02

*MCMO, Multiple-Choice Model for Omissions; 3PL-IAS, 3PL with Incorrect Answer Substitution; B-MIRT_MC_, Between-Item Multi-dimensional Model with multiple-choice items (Holman and Glas, 2005)*.

#### 5.2.3. Validity of Ability Estimates

The reliability of the ability estimates for the constructed-response items under the GPCM-IAS were 0.78 and 0.71 in mathematics and physics, respectively. The reliability of the ability estimates for the constructed-response items under the B-MIRT_CR_ were 0.76 and 0.69 in mathematics and physics, respectively. The correlations between the latent ability and the propensity to omit in the B-MIRT_CR_ were −0.52 in the mathematics items and −0.59 in the physics items, which indicates a clear MNAR mechanism. The results of the hierarchical regression models for the convergent validity between the multiple-choice and constructed-response items are presented in Table 6.

**Table 6:** Results for the hierarchical regressions between the EAP estimates from the constructed-response items and from the multiple-choice items with three different IRT models.

**DV**	**Test content and predictors**	***B*_1_**	***B*_2_**	***R*^2^**	***R*^2^-change**	***F*-change**	***df*_1_**	***df*_2_**
${\hat{\theta }}_{\text{GPCM}-\text{IAS}}$	**Mathematics**							
	${\hat{\theta }}_{3\text{PL}-\text{IAS}}$	0.76^**^	-	0.58	0.58	8, 392.9^**^	1	5,964
	${\hat{\theta }}_{3\text{PL}-\text{IAS}}$,${\hat{\theta }}_{\text{MCMO}}$	0.08^*^	0.69^**^	0.60	0.02	305.5^**^	2	5,963
	$\hat{\theta }$_B_−MIRT_MC_	0.75^**^	-	0.56	0.56	7, 563.8^**^	1	5,964
	$\hat{\theta }$_B_−MIRT_MC_, ${\hat{\theta }}_{\text{MCMO}}$	0.04	0.74^**^	0.60	0.04	686.4^**^	2	5,963
	**Physics**							
	${\hat{\theta }}_{3\text{PL}-\text{IAS}}$	0.60^**^	-	0.36	0.36	2, 256.5^**^	1	4,076
	${\hat{\theta }}_{3\text{PL}-\text{IAS}}$, ${\hat{\theta }}_{\text{MCMO}}$	0.07	0.55^**^	0.38	0.02	165.1^**^	2	4,075
	$\hat{\theta }$_B_−MIRT_MC_	0.58^**^	-	0.34	0.34	2, 095.9^**^	1	4,076
	$\hat{\theta }$_B_−MIRT_MC_, ${\hat{\theta }}_{\text{MCMO}}$	−0.01	0.63^**^	0.38	0.04	272.8^**^	2	4,075
${\hat{\theta }}_{\text{B}-\text{MIR}{\text{T}}_{\text{CR}}}$	**Mathematics**							
	${\hat{\theta }}_{3\text{PL}-\text{IAS}}$	0.76^**^	-	0.58	0.58	8, 263.4^**^	1	5,964
	${\hat{\theta }}_{3\text{PL}-\text{IAS}}$,${\hat{\theta }}_{\text{MCMO}}$	0.06	0.70^**^	0.60	0.02	324.7^**^	2	5,963
	$\hat{\theta }$_B_−MIRT_MC_	0.75^**^	-	0.57	0.57	7, 814.5^**^	1	5,964
	$\hat{\theta }$_B_−MIRT_MC_, ${\hat{\theta }}_{\text{MCMO}}$	0.12^**^	0.65^**^	0.60	0.04	545.6^**^	2	5,963
	**Physics**							
	${\hat{\theta }}_{3\text{PL}-\text{IAS}}$	0.60^**^	-	0.35	0.35	2, 217.3^**^	1	4,076
	${\hat{\theta }}_{3\text{PL}-\text{IAS}}$, ${\hat{\theta }}_{\text{MCMO}}$	0.08^**^	0.52^**^	0.38	0.03	155.8^**^	2	4,075
	$\hat{\theta }$_B_−MIRT_MC_	0.57^**^	-	0.34	0.34	2, 062.2^**^	1	4,076
	$\hat{\theta }$_B_−MIRT_MC_, ${\hat{\theta }}_{\text{MCMO}}$	0.00	0.60^**^	0.38	0.04	259.2^**^	2	4,075

DV, Dependent variable; *p < 0.05; *p < 0.001.

The regression models including only the B-MIRT_MC_ scores as independent variable had the lowest *R*^2^. The increments in *R*^2^ obtained with the inclusion of the MCMO scores in the models were significant, regardless of the model used for the constructed-response items, representing an increase in shared variance from around 2 to 4% and part correlation coefficients from 0.14 to 0.21 (*p* < 0.05). Given the similarity of the results using the GPCM-IAS and the B-MIRT_CR_ displayed in Table 6, further results will only be shown for the GPCM-IAS, since it is the model used in the TIMSS scoring procedure.

**Figure 3:**
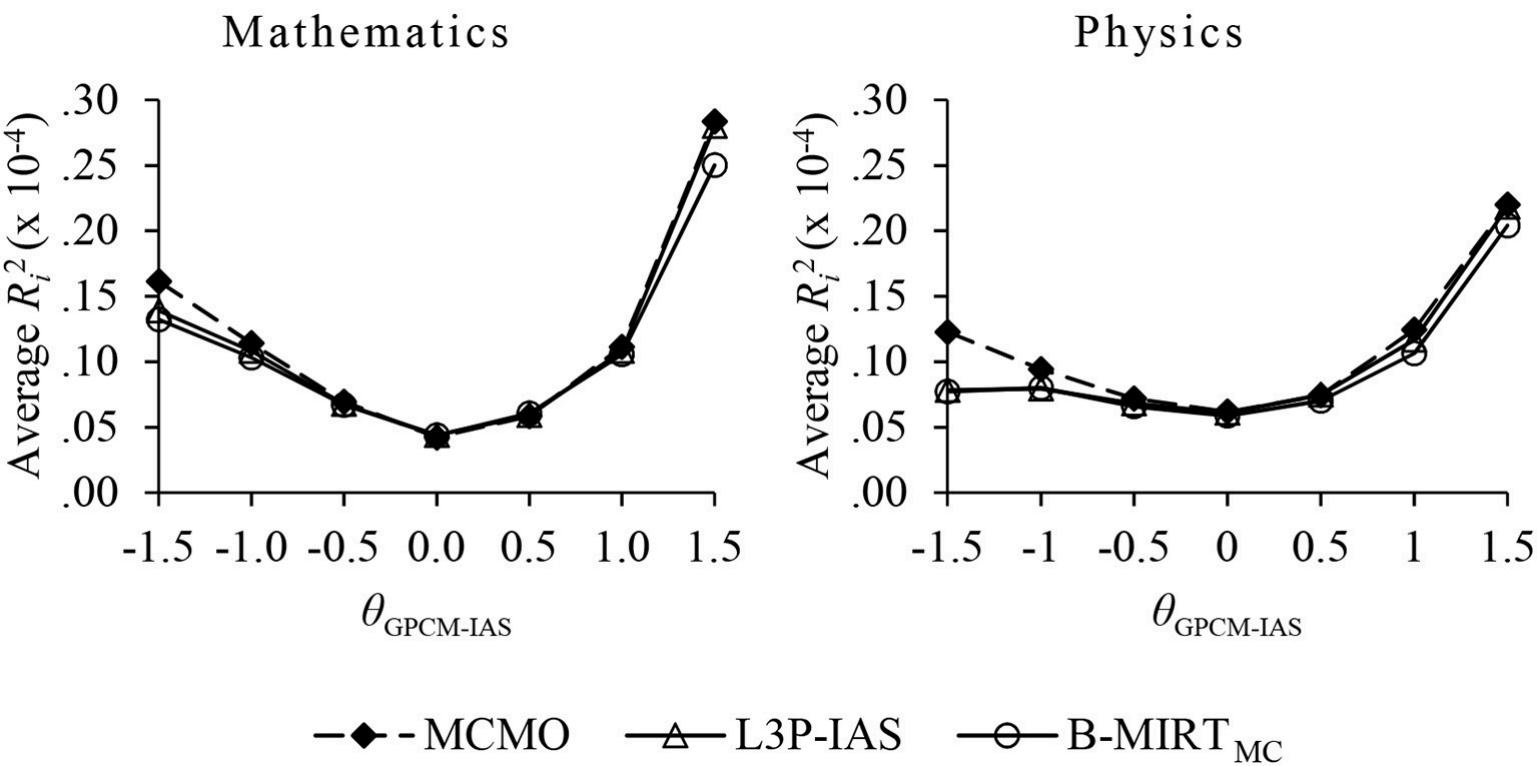
The expected contribution to *R*^2^ of a single examinee as a function of the ability estimates in the constructed-response items.

Figure 3 shows that the average examinee contribution to *R*^2^ varies across ability scores. The values in the vertical axis represent the expected contribution of a single examinee in each level of ${\hat{\theta }}_{\text{GPCM}-\text{IAS}}$. As expected, the greatest advantage of the MCMO over the 3PL-IAS and the B-MIRT_MC_ occurs for subjects with a low ability, since they are most likely to be in the DK state.

**Figure 4:**
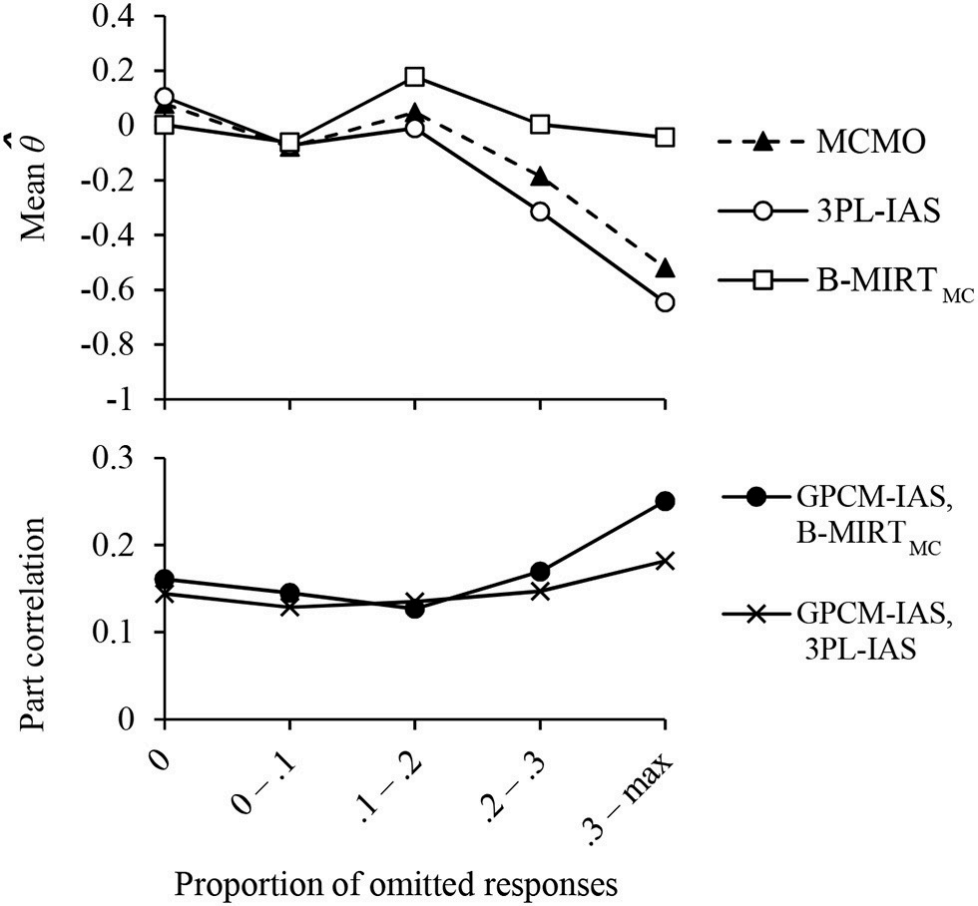
Average of the Mathematics ability estimates **(Top)**, and incremental validity of the MCMO Mathematics scores **(Bottom)** for groups with different proportions of omitted responses.

Figure 4 illustrates the difference of the ability estimates under the three models as a function of the proportion of omissions. The difference of the means between 3PL-IAS and MCMO ability estimates were found to be statistically significant for subjects with proportions of omitted responses of more than 0.2. The θ estimates under the B-MIRT_MC_ were significantly different from those from the other models for proportions of omitted responses higher than 0.1. The part correlations between the MCMO scores and the GPCM-IAS scores in the constructed-response items after controlling the variance explained by the B-MIRT_MC_ and by the 3PL-IAS in each group are presented in the bottom of Figure 4. The results show a pattern of incremental validity which is consistent with the difference in the ability estimates averages, suggesting that the MCMO scores differ from the ones obtained with the other models and that they are less biased than the others as the proportion of omitted responses increase.

Since the 3PL-IAS includes only the ability trait, the estimation of its pseudo-guessing parameter cannot address the variation in the lower ability asymptote caused by the propensity to omit showed in Figure 1C. The maximum likelihood estimate of the pseudo-guessing parameter of the 3PL-IAS will take the value that maximizes the model likelihood given the observed data, and therefore it will tend to reflect the probability of guessing where the density of ξ is maximum. If the mean of the propensity to omit is low, as found in TIMSS Advanced 2015 data, the estimate of the pseudo-guessing parameters will be similar to the expected by the MCMO.

As can be seen in the figure, the probability of responding correctly for the examinees with low abilities and high propensity to omit will be asymptotic to zero, given that they are more likely to omit. Since the ML estimate of the pseudo-guessing parameter reflects mostly the guessing probability for subjects with low ξ (where the density of ξ is maximum in the data used) and predicts lower asymptote higher than zero, the proficiency scores for examinees with low θ and high ξ will be underestimated.

On the other hand, the B-MIRT_MC_ ability scores were systematically overestimated for most groups of proportions of omitted responses. As indicated previously, by not accounting for guessing in the B-MIRT_MC_, a correct guess may be attributed to having a certain level of knowledge, which can lead to overestimated abilities of the less proficient examinees.

Although the models significantly differ for the ability estimates of the examinees with a missing proportion of more than 0.1 or 0.2, no differences in the estimation of the ability scores between the models were found in the country-level comparison. This may occur because most of the subjects had no omitted responses.

#### 5.2.4. Consistency of Propensity to Omit Estimates

**Figure 5:**
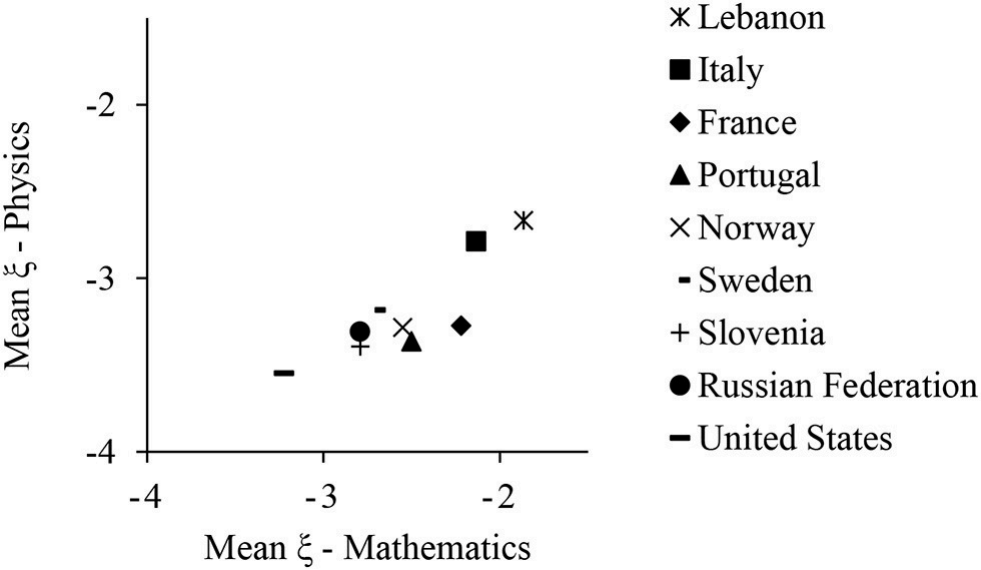
Country averages of the propensity to omit in TIMSS Advanced 2015 under the MCMO.

Figure 5 depicts the averages of ξ in mathematics and physics for each country and reflects a clear positive tendency. The correlation between country averages on the propensity to omit in the different test contents had a magnitude of 0.86 (*p* < 0.01). This indicates that the between-country differences in propensity to omit were consistent across the test contents.

## 6. Discussion

The main objective of this article was to present a new two-dimensional model accounting for omissions in multiple-choice items, analyzing the accuracy of its estimates under different testing conditions, and comparing it with two common approaches: Holman and Glas' Between-Item Multi-dimensional IRT model and the 3PL model with incorrect answer substitution for omissions. The proposed model extends the Multiple-Choice Model originally proposed by Samejima (1979) and Thissen and Steinberg (1984) by adding a probability to omit which is conditional on being in a *don't know* latent state, which is governed by a latent propensity to omit. Its theoretical formulations are based on behavioral assumptions about the omission process and has similarities with the rationale proposed by Lord (1983), with some differences on the specification of the functions for having a preference and choosing a response, and enabling the estimation of the pseudo-guessing parameter.

The accuracy of the recovery of the MCMO parameters was encouraging. In general, the estimation of scale and position parameters was highly precise in all simulated sample sizes, test lengths and expected proportions of omissions. Although the correlations between the true and the estimated *d*′ parameters were reasonably low, these innaccuracies appeared to have a negligible effect on the estimation of the low ability levels. Furthermore, the RISE values were good, being similar to the observations made using other unidimensional dichotomous models (Chen and Thissen, 1999). The ability levels were also well recovered in all conditions.

Conversely, the accuracy of the estimation of the propensity to omit appeared to strongly depend on the ability level of the examinees, the number of items and the percentage of omissions in the data. In a separate analysis using simulees with the 20% lowest θ and 20% highest θ, the ξ estimates were considerably more accurate for the first group. This suggests that the propensity to omit may be reliable for the less proficient examinees, but not for those with moderate or high ability. Unfortunately, this problem may be inherent to omitted responses in general, since as proficiency increases, less subjects will omit given that they may think that they know the answers. Therefore, the propensity to omit will be imprecise whenever there are few omitted responses, regardless of the model used. One possible solution for this problem may be to use the MCMO with items from different scales, as, for example, a set of items measuring Reading and another set measuring Science, and assuming a single propensity to omit underlying the response process to all the items. In this case, the examinees that excel in one scale may not be as proficient in the other scale, so it would be more likely for them to be in the DK state and decide to omit some items. Assuming a multivariate normal distribution of the ability traits and given its density function, as the correlation between abilities gets lower, the expected proportion of examinees with high levels of both ability traits is smaller. Furthermore, given that the precision of ξ depends on an examinee's proportion of omitted responses and that the later depends on the probability of being in the DK state (which is a function of ability), if the correlation between abilities is low, fewer examinees are likely to be in DK in the items of both scales. Either way, the interpretations about the propensity to omit must be made carefully and it is advisable to inform the standard error of the estimates.

The analyses with the TIMSS Advanced 2015 data offered moderate to high reliabilities of the MCMO ability scores in both mathematics and physics. Differently, as initially suggested by the results on the parameter recovery, the conditional reliability of ξ was acceptable mainly at low abilities and central propensities to omit. The fitness superiority of the MCMO vs. the 3PL-IAS and the B-MIRT_MC_ was noteworthy, since the χ^2*^/*df* ratios for the MCMO were below three in all of the items. By contrast, the 3PL-IAS offered the worst results, with 5 to 6 items heavily misfitted. The application of the MCMO offered significant increments in the convergent validity between the scores from the multiple-choice and constructed-response items, with an increase of around 0.02 to 0.04 in *R*^2^. As shown in Figure 3, these increments seemed to be higher for those subjects with low abilities. Finally, the high correlation between the country means of ξ in the mathematics and the physics items suggest that (1) the propensity to omit is somehow related to the country of residence of the examinees, and (2) the propensity to omit is independent of the test contents. In general, the MCMO offered good psychometric properties and proved to be superior to both the 3PL-IAS and the B-MIRT_MC_ with real data.

One main assumption of the MCMO is that the examinees deliberately decide whether to omit or not once they consider they do not know the answer. For this to be true, examinees must pay attention to the statements and fully process each item. An important limitation of the second study of this article is the possible presence of subjects with low motivation toward the assessment, which may affect the validity of our interpretations (Finn, 2015). This limitation is common to the studies with low stakes testing, and there is still no consensus on how to address it. Recent studies have found that more than 20% of the subjects may respond with little effort, engaging in rapid guessing, not giving enough thought to the items or not reaching the end of the test (Hoyt, 2001). Low-motivation behaviors can lead to an underestimation of what a student actually knows (Wise et al., 2006) and may bias the psychometric properties of the test scores, such as underestimating convergent validity and overestimating the internal consistency (Wise et al., 2009). To improve the construct validity, some studies suggest filtering out unmotivated examinees based on their responses to self-report motivation questionnaires or on their response times (Wise and Kong, 2005; Finn, 2015). At the time, however, no study has investigated how motivation affects omitted responses. Given that TIMSS Advanced 2015 does not include any of these measures, we were not able to investigate how the low motivation may affect the validity of the results of this article. Future studies may consider analyzing, for example, the time dedicated in omitting as an indicator of whether examinees fully process the items they skip.

This study opens various possibilities for future research. Further investigations should be carried out to analyze to what extent these results can be generalized, for example, for items with more than three alternatives, for more than one ability trait or for different assumptions about the trait distributions (e.g., Köhler et al., 2015; Rose et al., 2017). Also, treatments of the not-reached items were not within the scope of this article and therefore were not considered. Further modifications of the MCMO can allow the inclusion these indicators, as, for example, using of the examinees' count or proportion of not-reached items as a predictor of θ and ξ in a latent regression model (e.g., Rose et al., 2010, 2017; Pohl et al., 2014).

## Data Availability Statement

The international TIMSS Advanced 2015 datasets analyzed in this study can be found on the TIMSS 2015 International Database webpage (https://timssandpirls.bc.edu/timss2015/advanced-international-database/) or on the IEA Study Data Repository (http://www.iea.nl/data.html).

## Author Contributions

All the authors have made substantial intellectual contributions to this study. The original idea for the MCMO was proposed by FA. The data simulations, analyses and the subsequent writing of this article have been carried out by RK under the supervision of FA and VP. FA and VP have reviewed the manuscript and gave critical comments. All authors approved the final version of the manuscript.

### Conflict of Interest Statement

The authors declare that the research was conducted in the absence of any commercial or financial relationships that could be construed as a potential conflict of interest.
