# Supplementary material for: A Two-Dimensional Multiple-Choice Model Accounting for Omissions
Source: Front Psychol. 2018 Dec 11;9:2540. doi: 10.3389/fpsyg.2018.02540 (PMC6297845; doi:10.3389/fpsyg.2018.02540)
Supplement: Supplementary file 1 [file Data_Sheet_1.PDF]

Supplementary Material: A two-dimensional multiple-choice model accounting for omissions

MCMO ESTIMATION ROUTINE

Estimation routine for the Multiple-Choice Model for Omissions using the *mirt* package in *R*.

```
library("mirt")

# MCMO response probability function

P.MCMO <- function(par, Theta, ncat){

        A <- par[1 : ncat]
        C <- par[(ncat + 1) : (2 * ncat)]
        d <- exp(par[(2 * ncat + 1) : length(par)]) / sum(exp(par[(2 * ncat + 1) : length(par)]))
        D <- matrix(rep(c(1,d), nrow(Theta)), nrow(Theta), byrow = TRUE)

        theta <- Theta[, 1]
        xi <- Theta[, 2]

        # Indicator of whether a category is an item alternative or an omission
        obs.cat <- matrix(c(0, rep(1, ncat - 1)), nrow(Theta), ncat, byrow = TRUE)

        # Probability of omiting in DK
        w <- 1/(1 + exp(-xi))

        z <- matrix(0, nrow(Theta), ncat)

        for(i in 1 : ncat)
                z[, i] <- A[i] * theta + C[i]

        # Probability of the latent states
        P.u <- exp(z) / rowSums(exp(z))

        # Probability of the response categories
        P.x <- (obs.cat * P.u + P.u[,1] * abs(obs.cat - w) * D)

        return(P.x)
}
```

```
# Model for 3−alternative items with omissions coded as the lowest category

par <− c(a0 = −2, a1 = −1, a2 = −1, a3 = 0, c0 = 0, c1 = 0, c2 = 0, c3 = 0, d1.prime = 0, d2.prime = 0,
    d3.prime = 0)

est <− rep(TRUE, length(par))

MCMO.item <− createItem("MCMO.3", par = par, est = est, P = P.MCMO)

model.MCMO <− paste0("F1 = 1−", ncol(dat), "
        F2 = 1−", ncol(dat), "
        FIXED = (1−", ncol(dat), ", a3),
                (1−", ncol(dat), ", c3),
                (1−", ncol(dat), ", d3.prime)
        PRIOR = (1−", ncol(dat), ", d1.prime, norm, 0, .5),
                (1−", ncol(dat), ", d2.prime, norm, 0, .5)
        COV = F2*F2
        MEAN = F2")

mirt.MCMO <− mirt.model(model.MCMO)

mod <− mirt(dat, mirt.MCMO , "MCMO.3", customItems = list(MCMO.3 = MCMO.item))

trait.est <− fscores(mod, method = "EAP")
```
